# Dementia care and service systems – a mapping system tested in nine Swedish municipalities

**DOI:** 10.1186/s12913-018-3592-x

**Published:** 2018-10-16

**Authors:** Connie Lethin, Lottie Giertz, Emme-Li Vingare, Ingalill Rahm Hallberg

**Affiliations:** 1 Faculty of Medicine, Department of Health Sciences, Lund University, PO Box 157, 221 00 Lund, Sweden; 2 Department of Social Work, Linnaeus University, Universitetsplatsen 1, 351 95 Växjö, Sweden; 3 Faculty of Medicine, Department of Clinical Sciences, Clinical Memory Research Unit, Lund, Sweden

**Keywords:** Dementia, Chain of care, Screening, Institutional care, Palliative care, Professional care providers, Education

## Abstract

**Background:**

In dementia care, it is crucial that the chain of care is adapted to the needs of people with dementia and their informal caregivers throughout the course of the disease. Assessing the existing dementia care system with regard to facilities, availability and utilization may provide useful information for ensuring that the professional dementia care and service system meets the needs of patients and their families from disease onset to end of life.

**Methods:**

The aim of this study was to further develop and test a mapping system, and adapt it to a local context. In addition, the aim was to assess availability and utilization of care activities as well as professional providers’ educational level in nine municipalities under the categories of *Screening, the diagnostic procedures, and treatment*; *Outpatient care facilities*; *Institutional care* and *Palliative care*. This cross-sectional study was conducted in April through May 2015. Data was derived from the health care and social service systems in nine rural and urban municipalities in two counties in Sweden. The mapping system covered seven categories with altogether 56 types of health care and social service activities.

**Results:**

The mapping system was found to be reliable with minor adaptations to the context mainly in terms of activities. Availability of care activities was common with low utilization regarding *Screening, the diagnostic procedures, and treatment; Outpatient care facilities*; *Institutional care* and *Palliative care* and dementia trained staff was rare. Availability and utilization of care activities and professionals’ educational level was higher concerning *screening, the diagnostic procedures and treatment* compared with *outpatient care facilities*, *institutional care* and *palliative care*.

**Conclusions:**

The mapping system enables policy makers and professionals to assess and develop health care and social service systems, to be offered proactively and on equal terms to people with dementia and their informal caregivers throughout the course of the disease. The educational level of professionals providing care and services may reveal where, in the chain of care, dementia-specific education for professionals, needs to be developed.

## Background

In dementia care, it is crucial that the chain of care is adapted to the needs of people with dementia and their informal caregivers throughout the course of the disease. Dementia diseases are progressive and individuals at different stages have different needs in terms of professional care and service, including support and knowledge transfer to informal caregivers. Currently there is a lack of knowledge about how to best structure health care and social service systems to meet the needs throughout the disease process. A newly developed mapping system to assess dementia care systems was applied at a national level in eight European countries in the RightTimePlaceCare (RTPC) study (No. 24 21 53), conducted between 2010 and 2013 [1]. So far, it has not been tested locally in municipalities and counties. Assessing the dementia care system regarding facilities, availability and utilization may be informative and serve to ensure that the professional dementia care and service system meets the needs of patients and their families from disease onset to end of life.

With the demographic transition to an ageing population taking place worldwide, more people are developing dementia [2]. The political view in Western societies has been that people should be able to “age in place” [3] i.e. at home, informal caregivers more transferring more responsibility to informal caregivers. Thus, people with dementia will more commonly be cared for at home, with an informal caregiver involved as the main provider of care and services. Dementia diseases progress towards increased dependence in activities of daily living as well as severe communication difficulties, in particular at the later stage of the disease. This may be experienced as being out of control, as well as feelings of uncertainty, and fear of the unknown [4] and thus trigger behavioural problems. People with dementia and their caregivers sometimes experience professionals as mainly reactive to their needs [5], and perceive the dementia care system as fragmented and hard to access [6, 7].

This challenges the professional health care and social service system to deliver a continuum of individualized care and support for people with dementia and their informal caregivers [8, 9]. It needs to include information about available professional care and services, knowledge about how to provide basic care, about the dementia disease and how to understand and meet the needs of the person with dementia, and respite care for both people with dementia and their informal caregivers. Access to formal care, i.e. availability and utilization of health care and social services, is dependent on professionals’ and informal caregivers’ ability to interact with the accessibility [10] and, in addition, the professionals’ educational level.

This study reports on a Swedish project titled, “Living with dementia, care and service systems (LWD)” (No. 2013121), which was inspired by the RTPC study. Instead of taking a national perspective, a local perspective was adopted when applying the study to municipalities, to explore the variation between municipalities and test the mapping system for its applicability in illuminating resources available to people with dementia diseases and their informal caregivers. It is hoped that through the mapping system, the municipalities will be able to critically review and adjust the system to serve people with dementia and their caregivers. The LWD study addresses key issues in dementia care such as the range of care and services, living conditions for people with dementia and informal caregivers, and their quality of life, applying a model based on the one hand, on the process of dementia from early stage to end of life and on the other hand, the type of activities needed to meet the needs of them.

The original mapping system [1] aimed to get a national overview of available and utilized care activities in eight European countries, and covered 50 types of health care and social services (from here on referred to as “care activities”) categorized in seven categories throughout the course of the dementia disease. Furthermore, the aim was to explore who professional caregivers educational level according to the International Standard Classification of Education (ISCED) [11] for each care activity. The mapping system was originally developed by researchers from each country, identifying different activities provided by professionals and non-professionals. Agreement on concepts and terminology was worked through, inspired by consensus methods [12].

The original mapping system covered a wide range of different care activities and these were structured into seven care categories: *Screening, the diagnostic procedures, and treatment*, and *Outpatient care facilities*. Furthermore, they covered *Care at home*, *Institutional care*, *Palliative care*, *Informal caregiving and supportive actions*, and *Civic organizations*. The categories was based on knowledge about needs during the process of dementia. Most of the care activities were in the category *Care at home* (*n* = 18) and the least range of activities were in the category of *Informal caregiving and supportive actions* (*n* = 4) [1]. Analysing the *Care at home* category showed higher availability than utilization [13], and the findings indicated more similarities than differences between the eight European countries. Furthermore, the original study revealed that specialized dementia care and services were sparsely available and even more sparsely utilized [1]. Support for informal caregivers was available but utilized by few and this was consistent for all the countries. Several types of professionals were involved in the care activities, but it was not always clear who was responsible for what [14] and the more personal care required the lower the education of the professionals involved. Those involved in *Screening, the diagnostic procedures, and treatment* and *Outpatient care facilities* were educated at a Bachelor’s level or higher, while everyday care was provided by professionals, trained at a lower ISCED level or workers with no formal training at all. Professionals specifically trained in dementia care were rare [14, 15]. In addition, countries with national guidelines seemed more aware of the importance of professionals with specialized dementia education [15].

The rationale behind this study is that testing and developing the mapping system further may be useful for assessing the care activities at the local level, including the structure of the health care and social service systems. Furthermore, it may be useful for describing availability and utilization of health care and social services for people with dementia and their informal caregivers and for determining professional providers’ educational level. By making the resources available and visible to the staff as well as the informal caregiver and the person with dementia it may be used more efficiently, some activities taken out and others added. Policy makers and professionals may be enabled to develop their health care and social service system. Making the system visible to professionals, they can be more proactive and communicate available care and support to people with dementia and caregivers. This is important to ensure transparency, accessibility, and equal and individualized health care and social services to all citizens regarding dementia care, and also to fill the gaps and systematize the chain of care in accordance with the disease progression.

## Methods

### Aims

The aim of this study was to further develop and test the mapping system in a local context of nine Swedish municipalities. In addition, the aim was to assess availability and utilization of care activities as well as professional providers’ educational level under the categories of *Screening, the diagnostic procedures, and treatment*, *Outpatient care facilities*, *Institutional care* and *Palliative care*.

### Context

Health care in Sweden is performed in accordance with the Health and Medical Services Act [16] regulating the responsibility for health care across 21 counties and 290 municipalities. The county councils are responsible for health care, i.e. assessment and treatment, in hospitals and outpatient clinics. The municipalities are responsible for care and social services, i.e. home care and services, day care and nursing homes. Care for individuals aged 65 years and older and living at home, is granted after needs assessment, according to the Swedish Social Services Act [17].

### Design and sample

This was a cross-sectional descriptive study of the care and services available to people with dementia and their informal caregivers throughout the course of the disease. The study population was nine municipalities (1–9) in two counties in southern Sweden. Data was collected during April to May 2015, by people knowledgeable about their municipality. Each municipality selected one or two contact persons representing the municipality. One municipality nominated three contact persons to have back-up in case of absences. These were professional providers such as registered nurses specialized in dementia, social workers and care coordinators with experience of dementia care. The researchers and contact persons had regular meetings, training and monitoring to discuss the description of the care activities and professionals, the meaning of availability and utilization and how to use the mapping system.

In all there were 220,641 inhabitants in the municipalities, 21% of whom were aged 0–17, 58% who were aged 18–64, and 21% who were 65 years or older. The number of inhabitants varied from 1944 to 88,108, and three of the nine municipalities (municipalities 2, 5 and 6) represent rural areas. People with a foreign background accounted for 24.1% (mean) (range 21.4–26.8%) of these municipal populations, compared with 22.2% for Sweden overall. In 2014, the nine municipalities together had 1087 persons with a dementia diagnosis [18].

### The mapping system

The original mapping system horizontally explored five stages of the dementia disease – the diagnosis stage; early stage; intermediate stage; late stage; and end-of-life [19]. Vertically, 50 different types of care activities were listed and defined and they were based on how the disease progressed over time as reflected in type of care needs. Each aspect of the care activities included estimations of availability, utilization, and providers of care and service, at each stage of the dementia disease. This division in availability versus utilization was based on that availability can be common whilst in fact it is rarely used for various reasons. Response alternatives for estimations of availability were “For all”, “For most”, “For few” and “For no one”; and estimations of utilization were “By all”, “By most”, “By few”, and “By no one”. Professional care providers were classified regarding educational level in accordance with the ISCED [11] and were reported for each care activity [1].

The mapping system was adapted to the local context, in consensus with the researchers and contact persons in each municipality (Table 1). Each care activity was thoroughly.

**Table 1:** Care activities in the mapping system with newly added or; removed activities and refined description of activities

Care activities in the original mapping system	Care activities in the developed mapping system
Screening, the diagnostic procedures, and treatment	Screening, the diagnostic procedures and treatment
Dementia screening programme/“case finding”	No changes
Standard diagnostic procedure for dementia disease	No changes
Pharmacological treatment specific for dementia disease	No changes
Non-pharmacological treatment	No changes
Pharmacological treatment specific for BPSD	No changes
Non-pharmacological treatment specific for BPSD	No changes
Memory clinic	No changes
Outpatient care facilities	Outpatient care facilities
Outpatient clinic specific for dementia diseases	Newly added activity - Specialized team in primary care for PwD
Counselling	No changes
Day care/Day activity/Day Care Centre/Day hospital	No changes
Day care/Day activity/Day Care Centre/Day hospital specialized in dementia care	No changes
Care at home	Care at home
Will be presented elsewhere	One activity removed and will be presented elsewhere
Institutional care	Institutional care
Rehabilitation in institution	Newly added activity – Safety accommodation for people 70+
Residential home/Sheltered home/Assisted living	Newly added subactivity^a^ – Respite care, temporary and in case of emergency
Nursing home, for older people in general	Newly added subactivity^a^ – Respite care, specialized in dementia care, temporary and in case of emergency
Nursing home, specialized in dementia care	
Nursing home, with dementia care units	Refined description – Community dwelling / Small housing for
Group dwelling/Small scaled living/Dementia patients’ house unit	people with dementia
Behavioural Intensive Care Unit/Psychogeriatric unit/ Geriatric psychiatry inpatient unit	Refined description – Psychogeriatric unit/Geriatric psychiatry inpatient unit
Respite care	Refined description – Respite care, temporary and planned
Respite care, specialized in dementia care	Refined description – Respite care, specialized in dementia care, temporary and planned
Palliative care	Palliative care
Hospice/Institutional palliative care	No changes
Hospice/Palliative care at home	No changes
Advanced directive	No changes
Informal caregiving and supportive actions	Informal caregiving and supportive actions
Will be presented elsewhere	Will be presented elsewhere
Civic organizations	Civic organizations
Will be presented elsewhere	Two activities removed and will be presented elsewhere

*BPSD* Behavioural psychological symptoms in dementia, *GPS* Global positioning system, *IADL* Instrumental activities in daily life, *PADL* Personal activities in daily life, *PwD* Person with dementia

^a^existing activities subdivided into smaller parts

discussed, as were the professional providers and their educational level according to the ISCED [11], until agreement was reached (Table 2). Where needed, specific professional

**Table 2:** Professional care providers’ educational level in dementia care in Sweden, according to ISCED [11]

ISCED LEVEL:	General health care training	Specialized health care training	Specialized training in dementia
7: Master’s or equivalent, vocational	Dentist (DE) investigates, treats diseases and injuries in the teeth and jaws. The treatment can range from prevention and disease treatment, to extensive and technologically advanced clinical interventions. Dietician (DI) provides nutritional counselling, prevention and treatment Medical doctor (MD), investigate, treat and prevent diseases and other health problems at hospitals and health centres, in the pharmaceutical industry or occupational health services. Psychologist (psychol) provides counselling and help to people with psychological problems. Physiotherapist (PT) provides rehabilitation to identify and improve, e.g., disabled movement and function. Social worker (SW) provides staff management for residential care or home help service. Speech therapist (ST) treat communication problems due to language, speech and voice disturbance including diagnostics and treatment of swallowing disorders.	General practitioner (GP) physician who treats patients within a district for all types of diseases. Medical doctor-internal medicine (MD-int) specialized in internal medicine. Medical doctor-neurology (MD-neuro) specialized in neurology. Training in dementia is normally part of their special training. Medical doctor-psychiatry (MD-psych)/Old Age Psychiatrist specialized in psychiatry. Training in dementia is normally part of their special training. Psychologist-neuro (psychol-neuro) psychologists specialized in cognitive neuroscience, behaviour and brain function. Administer neuropsychological tests.	Medical doctor-geriatrics (MD-ger) geriatrician or psycho- geriatrician specialized in geriatrics. Occupational therapist (OT-dem) Specialized in dementia (1 year Master). Physiotherapist (PT-dem) is specialized in dementia (1 year Master). Registered nurse (RN dem) has the overall responsibility for dementia care in an area/municipality. Provides counselling, supervision, and assessments, and mediates contacts. Education at advanced level: Care of the elderly (1-year Master), District nurse (1-year Master), Psychiatric care (1-year Master), Dementia (1-year Master).
6: Bachelor’s or equivalent, vocational	Case manager (CM) see “Specialized health care training”. Dietician (DI) provides nutritional counselling, prevention and treatment. Occupational therapist (OT) provides rehabilitation to achieve optimum level of functional ability. This may include adaptation of the home and providing aids and equipment to assist with managing everyday activities. Registered nurse (RN) provides care and service including help with PADLs, medical treatments, and managing the nursing care team. Physiotherapist (PT) provides rehabilitation to identify and improve, e.g., disabled movement and function. Social worker (SW) provides staff management for residential care or home help service. Speech therapist (ST) treat communication problems due to language, speech and voice disturbance including diagnostics and treatment of swallowing disorders.	Case manager (CM) professional (RN or SW) function that may include finding and outreach, comprehensive assessment and care planning, coordination of service, service provision, monitoring, and evaluation, and, in addition, meeting special care needs Community psychiatric registered nurse (RN-comm-psych) supports older people at home and in nursing/residential homes. Specialized in psychiatry Home help officer (HO) carries out needs assessment prior to decision about home services and care	Occupational therapist (OT-dem) is specialized in dementia. Physiotherapist (PT-dem) is specialized in dementia. Registered nurse (RN dem) has an overall responsibility for dementia care in an area/municipality. Provides counselling, supervision, and assessments, and mediates contacts. Education at advanced level: Care of the elderly (1-year Master), Primary care (1-year Master), and Psychiatric care (1-year Master).
5: Short-cycle tertiary education, vocational	Dental hygienist (DH) helps prevention by brushing and polishing the teeth as well as removing plaques. A dental hygienist may diagnose and treat caries, inflammation and dental loss.		State examined nurses specialized in dementia care (SEN dem) not Bachelor’s level.
4: Post-secondary non-tertiary, vocational	Licensed practical nurse (LPN)/Auxiliary nurse (Aux-N) provides care and service including help with IADLs and PADLs, and, in addition, minor medical treatment. Health care trained at secondary school level.		
3: Upper secondary, vocational	Nurse aid/assistant nurse (Ass-N) provides care and service including help with IADLs and PADLs. Health care trained for < 6 months. Support worker (Supp-work) home carer, psychological supporter, or home trainer paid at enhanced nursing assistant/home carer rate. Social care/nursing trained at secondary level or trained on the job.		Caregiver coordinator (CC) a professional coordinating care for people with dementia and informal caregivers.
< 3	Occupational therapist assistant (OT-ass) performs similar tasks as the OT. Not trained, or trained on the job. Social worker assistant (SW-ass) performs similar tasks as the SW. Not trained, or trained on the job.		

*IADL* Instrumental activity of daily living, *ISCED* The International Standard Classification of Education, *PADL* Practical activity of daily living

providers (a social worker or care manager) were contacted for accurate information. Furthermore, information was gathered from the internet regarding professionals, and their levels of education and care training descriptions. Care activities were removed, remained unchanged or were refined in the description. After minor adjustment in the descriptions of care activities, the mapping system was pilot tested in two municipalities, No changes were made after the pilot testing so the mapping system was adapted in the other seven participating municipalities. Data was collected according to the manual developed for this study, concerning availability and utilization of care activities for dementia care, and providers for each care activity during the last 6 months by the contact persons.

### Analysis

Availability and utilization of care activities and the professionals’ educational level were analysed descriptively with regard to variation for each of the four stages of the dementia disease and in each of the nine municipalities. For all seven care categories included in the mapping system, response alternatives for availability and utilization of the 56 care activities were “For all” and “For most” and are given in bold in the Tables to distinguish from the response alternatives “For few” and “For no one”. Professionals involved in the care was recorded in a separate mapping system. The categories *Screening, the diagnostic, procedures and treatment*, *Outpatient care facilities*, *Institutional care* and *Palliative care* are described in this study. The three categories remaining, *Care at home*, *Informal caregiving and supportive actions* (including counselling and day care form Outpatient care facilities) and *Civic organizations,* will be reported elsewhere.

## Results

### The developed mapping system

When reviewing the care categories, all seven care categories were found to be valid and remained as in the original mapping system. A review was made of the stages of dementia as there were insignificant differences between estimations of availability and utilization in the diagnosis stage and the early stage. Consequently, the two stages were collapsed into one – the “early stage”. For further development of the mapping system and to apply it to the local context, as well as to the national guidelines [20] and laws regulating health care and the social service system [16, 17], four care activities were added and three were removed (Table 1). Five care activities were refined in the description and subdivided to give ten care activities. The mapping system therefore covered 56 care activities and was found to be reliable after minor adaptation to the context.

New care activities were added under the categories *Outpatient care facilities* (namely “specialized team in primary care for persons with dementia”); *Care at home* (“contact person”, in accordance with the Social Service Act and “respite care at home”) (not shown in Table 1); and *Institutional care* (“safety accommodation for people aged 70+”). The care activity “nursing home, with dementia care units” was removed from the category *Institutional care* because nursing homes in Sweden either are for older people in general or are specialized in dementia care. Under the category *Civic organizations*, two care activities were removed; “paid voluntary organization” and “self-help organization/self-support group”.

As previously mentioned, minor changes were made to refine the definition of four care activities which were then subdivided to give ten care activities (“voluntary surveillance by telephone” and “technical equipment”; “voluntary aid”; two activities, one with and another without specialization in dementia care, of “respite care, temporary and planned”; two activities, one with and another without specialization in dementia care, of “respite care, temporary and in case of emergency”). Provision of vision and hearing aids was changed to “own activities” as people 65 years and older can get remittance from healthcare for vision and hearing aids. Under the care category *Care at home*, “foot care” was removed as it was considered to constitute self-management unless the person was diabetic. When comparing the nine municipalities for availability of 19 care activities throughout the course of the disease, the mean availability was 13 care activities (range 11–16). Four municipalities offered more than 13 care activities and two municipalities, one rural and one urban, offered 16 out of 19 care activities.

Staff training and professionals’ educational level were added to apply to the Swedish health care and social service system. Concerning general health care training this was training to become a dentist, dietician, speech therapist, dental hygienist and occupational therapist assistant. Under “specialized health care training”, “psychologist specialized in neuroscience” was added, and regarding specialized training in dementia, occupational therapy and physiotherapy were added. The provider category of “care coordinator”, a professional coordinating care for people with dementia and their informal caregivers was added (Table 2).

Availability and utilization of care activities were higher for *Screening, the diagnostic procedures, and treatment* (Table 3) than for *Outpatient care facilities* (Table 4), *Institutional care* (Table 5) and *Palliative care* (Table 6). There were differences between availability and utilization of care activities in the nine municipalities, suggesting that the mapping system was able to assess and intercept variation. The educational level of professionals working in *Screening, the diagnostic procedures and treatment* (Table 7) was generally higher, mostly involving a Bachelor’s to a Master’s degree in Science compared with the educational level of professionals working in *Outpatient care facilities* (Table 8), *Institutional care* (Table 9) and *Palliative care* (Table 10).

**Table 3:** Availability and utilization of care activities in the category *Screening, the diagnostic procedures, and treatment*

Definition	Municipality*	Early stage		Intermediate stage		Late stage		End of life stage	
		Available for	Utilized by	Available for	Utilized by	Available for	Utilized by	Available for	Utilized by
Dementia screening programme/“case finding”: Carried out in primary care with the aim of detecting dementia at early stages. Different assessment tools might be used.	1	No one	No one	No one	No one	No one	No one	No one	No one
	2	All	All	All	Few	All	No one	All	No one
	3	No one	No one	No one	No one	No one	No one	No one	No one
	4	All	Few	All	Few	All	Few	All	No one
	5	No one	No one	No one	No one	No one	No one	No one	No one
	6	No one	No one	No one	No one	No one	No one	No one	No one
	7	All	Few	All	Few	All	Few	All	No one
	8	All	Most	All	Most	All	Most	All	Most
	9	All	Most	All	Most	All	Most	All	Most
Standard diagnostic procedure for dementia disease: National standard diagnostic procedure applied in accordance.	1	All	Most	All	Most	All	Few	All	No one
	2	All	All	All	All	All	Few	–	–
	3	All	Most	All	Most	All	Few	All	No one
	4	No one	No one	No one	No one	No one	No one	No one	No one
	5	All	Few	All	Most	All	Most	All	No one
	6	All	Most	All	Most	All	Few	All	No one
	7	All	Most	All	Most	All	Few	All	No one
	8	All	Most	All	Most	All	Most	All	Few
	9	All	Most	All	Most	All	Most	No one	No one
Pharmacological treatment specific for dementia disease: Medication of cognitive symptoms, combined with a likely slowing of disease progression, for instance Cholinesterase Inhibitors for improvement of cognition (memory, language). ATC code N06D [30]. Only validated treatment.	1	All	Most	All	Most	All	Few	All	No one
	2	All	All	All	Few	All	No one	No one	No one
	3	All	Most	All	Most	All	Few	All	No one
	4	All	Most	All	Most	All	Most	All	No one
	5	All	Few	All	Most	All	Most	All	Few
	6	All	Few	All	Most	All	Most	All	Few
	7	All	Most	All	Most	All	Few	All	No one
	8	All	Most	All	Most	All	Few	All	Few
	9	All	Few	All	Most	All	Most	All	Few
Non-pharmacological treatment: Treatment – not pharmacological, for instance orientation to reality, reminiscence therapy, tactile massage, cognitive stimulation etc.	1	Few	Few	Few	Few	Few	Few	Few	Few
	2	Most	Few	All	Most	All	Most	All	Few
	3	Few	Few	Few	Few	Few	Few	Few	Few
	4	All	Few	All	Most	All	Few	All	No one
	5	All	Few	All	Most	All	Most	All	Few
	6	All	Few	All	Few	All	Few	All	Few
	7	All	Few	All	Most	Most	Few	Few	Few
	8	All	Few	All	Few	All	Most	Most	Most
	9	No one	No one	No one	No one	No one	No one	No one	No one
Pharmacological treatment specific for Behavioural Psychological Symptoms of Dementia (BPSD): Pharmacological treatment of non-cognitive symptoms and behaviours, for instance psychotropic drugs for aggressive behaviour [31]. ATC code N05 [30].	1	All	Few	All	Few	All	Few	All	Few
	2	All	Few	All	Few	All	Most	All	Most
	3	All	Few	All	Most	All	Most	All	Few
	4	All	Most	All	Most	All	Most	All	Most
	5	All	Few	All	Few	All	Few	All	Few
	6	All	Most	All	Most	All	Most	All	Most
	7	All	Few	All	Few	All	Few	All	Few
	8	Most	Few	All	Most	All	Most	All	Most
	9	Most	Few	Most	Few	Most	Few	Most	Few
Non-pharmacological treatment specific for BPSD: Treatment – not pharmacological, for instance environmental modification, massage, presence of pets, selected music, distraction etc. [23].	1	Few	Few	Most	Most	Most	Most	Most	Most
	2	All	Few	All	Most	All	Most	All	Few
	3	Few	Few	Few	Few	Few	Few	Few	Few
	4	All	Few	All	Most	All	Most	All	Few
	5	Few	Few	Most	Most	Most	Most	Most	Most
	6	Most	No one	Most	Few	Most	Few	Most	Few
	7	Most	Few	Most	Few	Most	Few	Most	Few
	8	No one	No one	No one	No one	Few	Few	Few	Few
	9	Few	Few	Few	Few	Few	Few	Few	Few
Memory clinic: Outpatient clinic for examination and treatment of memory impairments, not only suspected dementia diseases.	1	All	Few	All	Few	All	Few	All	No one
	2	All	Few	All	Few	All	Few	All	No one
	3	Few	Few	Few	Few	Few	Few	No one	No one
	4	No one	No one	No one	No one	No one	No one	No one	No one
	5	Few	Few	Few	Few	Few	Few	No one	No one
	6	Few	No one	Few	Few	Few	Few	Few	No one
	7	All	Few	All	Few	All	Few	All	Few
	8	All	No one	All	No one	All	No one	All	No one
	9	No one	No one	Few	Few	Few	Few	No one	No one

*1–9 = represents the municipalities in two county councils in Sweden. *ATC* anatomical therapeutic chemical classification

*1–9 = represents the municipalities in two counties in Sweden

**Table 4:** Availability and utilization of care activities in the category *Outpatient care facilities*

Definition	Municipality^a^	Early stage		Intermediate stage		Late stage		End of life stage	
		Available for	Utilized by	Available for	Utilized by	Available for	Utilized by	Available for	Utilized by
Outpatient clinic specific for dementia diseases: Outpatient clinic in primary health care or in hospital, including care by physicians and nurses specialized in dementia.	1	No one	No one	No one	No one	No one	No one	No one	No one
	2	All	Few	All	Few	All	Few	All	Few
	3	Few	Few	Few	Few	Few	Few	Few	Few
	4	No one	No one	No one	No one	No one	No one	No one	No one
	5	No one	No one	No one	No one	No one	No one	No one	No one
	6	No one	No one	No one	No one	No one	No one	No one	No one
	7	Most	Most	Most	Most	Most	Few	Most	No one
	8	Few	Few	Few	Few	Few	Few	Few	Few
	9	No one	No one	No one	No one	No one	No one	No one	No one
Specialized team in primary care for persons with dementia: A team specifically for people with dementia with different professionals; medical doctors, registered nurses, occupational therapists, physiotherapist and licensed practical nurses.	1	Most	Most	Most	Most	Few	Few	No one	No one
	2	All	Most	All	Few	All	Few	All	Few
	3	Few	Few	Few	Few	Few	Few	Few	Few
	4	Few	Few	Most	Few	Most	Most	No one	No one
	5	No one	No one	No one	No one	No one	No one	No one	No one
	6	No one	No one	No one	No one	No one	No one	No one	No one
	7	No one	No one	No one	No one	No one	No one	No one	No one
	8	All	Few	All	Most	All	Most	All	Few
	9	All	Most	All	Most	All	Few	All	No one

^a^1–9 = represents the municipalities in two counties in Sweden

**Table 5:** Availability and utilization of care activities in the category *Institutional care*

Definition	Municipality^a^	Early stage		Intermediate stage		Late stage		End of life stage	
		Available for	Utilized by	Available for	Utilized by	Available for	Utilized by	Available for	Utilized by
Rehabilitation in institution: Training in residential home/nursing home/group dwelling by Occupational therapist, Physiotherapist and/or registered nurse and/or Licensed practical nurse/Auxiliary nurse to improve or maintain functional ability.	1	All	Few	All	Most	All	Most	All	Most
	2	All	Few	All	Few	All	Few	All	Few
	3	All	Few	All	Few	All	Most	All	Most
	4	All	Few	All	Few	All	Few	All	Few
	5	All	Few	All	Few	All	Few	All	Few
	6	All	Few	All	Few	All	Few	All	Few
	7	All	Most	All	Most	All	Most	All	Most
	8	All	Most	All	Most	All	Most	All	Most
	9	All	Most	All	Most	All	Most	All	Most
Safety Accommodation: Accommodation for people 70+, not specifically for people with dementia. The accommodation is staffed specific hours every day to offer joint meals and activities.	1	All	Few	All	Few	All	Few	All	No one
	2	No one	No one	No one	No one	No one	No one	No one	No one
	3	All	Few	All	Few	All	Few	All	Few
	4	No one	No one	No one	No one	No one	No one	No one	No one
	5	No one	No one	No one	No one	No one	No one	No one	No one
	6	No one	No one	No one	No one	No one	No one	No one	No one
	7	No one	No one	No one	No one	No one	No one	No one	No one
	8	Most	Few	Most	Most	Most	Most	Few	Few
	9	No one	No one	No one	No one	No one	No one	No one	No one
Residential home/Sheltered home/Assisted living. For older people not specific for those with dementia disease or mixed with other older people: Housing with care and service, i.e. home help services or the like for people who do not need daily attention from registered nurses or 24 h surveillance.	1	All	Few	All	Few	All	Few	All	Few
	2	All	Few	All	Most	All	Most	All	Most
	3	All	Few	All	Few	All	Most	All	Most
	4	All	Few	All	Few	All	Most	All	All
	5	All	No one	All	Few	All	Most	All	Most
	6	All	No one	All	Few	All	Most	All	Most
	7	All	Few	All	Few	All	Most	All	Most
	8	All	Few	All	Few	All	Most	All	Most
	9	All	Few	All	Most	All	All	All	Few
Nursing home. Not specific for those with dementia disease or mixed with other older people: Nursing care available 24/7 provided by employed staff. May include short-term rehab as well as long-term care for people with chronic impairments or disabilities requiring daily attention of registered nurses to help with personal care or mobility.	1	All	Few	All	Most	All	Most	All	Most
	2	All	No one	All	Few	All	Most	All	Most
	3	All	Few	All	Few	All	Most	All	Most
	4	No one	No one	Few	Few	All	Most	All	Most
	5	No one	No one	No one	No one	No one	No one	No one	No one
	6	No one	No one	No one	No one	No one	No one	No one	No one
	7	All	No one	All	Few	All	Most	All	Most
	8	No one	No one	No one	No one	No one	No one	No one	No one
	9	No one	No one	All	Few	All	Few	No one	No one
Nursing home with dementia care units: Nursing homes especially designed and adapted environment to meet the needs of people with dementia. Nursing care available 24/7 provided by employed staff. Units with staff specialized in dementia.	1	All	Few	All	Most	All	Most	All	Most
	2	No one	No one	No one	No one	No one	No one	No one	No one
	3	No one	No one	No one	No one	No one	No one	No one	No one
	4	No one	No one	No one	No one	All	Most	All	Most
	5	No one	No one	No one	No one	No one	No one	No one	No one
	6	No one	No one	No one	No one	No one	No one	No one	No one
	7	All	No one	All	Few	All	Most	All	Most
	8	No one	No one	No one	No one	No one	No one	No one	No one
	9	No one	No one	No one	No one	No one	No one	No one	No one
Community Dwelling / Small housing for people with dementia: Housing especially designed and adapted environment to meet the needs of people with dementia. Staff specifically trained in dementia care. Only for people with dementia (≤10 people)	1	All	Few	All	Most	All	Most	All	All
	2	All	No one	All	Few	All	Most	All	Most
	3	No one	No one	No one	No one	No one	No one	No one	No one
	4	No one	No one	No one	No one	No one	No one	No one	No one
	5	No one	No one	Few	Few	Few	Few	Few	Few
	6	All	No one	All	No one	All	Few	All	Few
	7	No one	No one	No one	No one	No one	No one	No one	No one
	8	No one	No one	No one	No one	No one	No one	No one	No one
	9	No one	No one	No one	No one	No one	No one	No one	No one
Psychogeriatric unit/Geriatric psychiatry inpatient unit: Units where patients are provided inpatient care during a short-intermediate period (weeks to some months), with the aim of controlling acute BPSD. Staff specialized in dementia management. Only people with dementia is signed in.	1	No one	No one	No one	No one	No one	No one	No one	No one
	2	No one	No one	Few	No one	Few	No one	No one	No one
	3	No one	No one	No one	No one	No one	No one	No one	No one
	4	–	–	–	–	–	–	–	–
	5	No one	No one	Few	Few	Few	Few	No one	No one
	6	No one	No one	No one	No one	No one	No one	No one	No one
	7	No one	No one	No one	No one	No one	No one	No one	No one
	8	Few	Few	Few	Few	Most	Most	Most	Most
	9	Few	No one	Few	Few	Few	No one	No one	No one

^a^1–9 = represents the municipalities in two counties in Sweden

**Table 6:** Availability and utilization of care activities in the category *Palliative care*

Definition	Municipality^a^	Early stage		Intermediate stage		Late stage		End of life stage	
		Available for	Utilized by	Available for	Utilized by	Available for	Utilized by	Available for	Utilized by
Hospice/Palliative care at home: Hospice is a type of care and a philosophy of care that focuses on the palliation of a terminally ill patient’s symptoms. The focus should be on physical, emotional, spiritual or social needs [23]. The care is provided at home.	1	All	Few	All	Few	All	Few	All	Few
	2	All	Most	All	Most	All	Most	All	Few
	3	All	Few	All	Few	All	Few	All	Few
	4	All	All	All	All	All	All	All	All
	5	All	Few	All	Few	All	Few	All	Few
	6	All	No one	All	Few	All	Few	All	Few
	7	All	Few	All	Few	All	Few	All	Few
	8	No one	No one	No one	No one	No one	No one	No one	No one
	9	All	Few	All	Few	All	Few	All	Few
Hospice/Institutional palliative care: Hospice is a type of care and a philosophy of care that focuses on the palliation of a terminally ill patient’s symptoms. The focus should be on physical, emotional, spiritual or social needs [23]. The palliative care is provided in hospitals or nursing homes.	1	All	Few	All	Few	All	Few	All	Few
	2	All	Most	All	Most	All	Most	All	All
	3	All	Few	All	Few	All	Most	All	All
	4	All	All	All	All	All	All	All	All
	5	All	Few	All	Few	All	Few	–	–
	6	All	No one	All	Few	All	Few	All	No one
	7	All	No one	All	Few	All	Most	All	All
	8	No one	No one	No one	No one	No one	No one	No one	No one
	9	All	Few	All	Few	All	Few	All	Few
Advanced directive: Living will and power of attorney.	1	All	Few	All	Few	All	Few	All	Few
	2	All	Few	All	Few	All	Few	All	Few
	3	No one	No one	No one	No one	No one	No one	No one	No one
	4	No one	No one	No one	No one	No one	No one	No one	No one
	5	Few	Few	Few	Few	Few	Few	Few	Few
	6	No one	No one	No one	No one	No one	No one	No one	No one
	7	No one	No one	No one	No one	No one	No one	No one	No one
	8	No one	No one	No one	No one	No one	No one	No one	No one
	9	No one	No one	No one	No one	No one	No one	No one	No one

^a^1–9 = represents the municipalities in two counties in Sweden

**Table 7:** Professional care providers level of education in the category *Screening, the diagnostic procedures, treatment of dementia*

		ISCED level 7: Master’s or equivalent, vocational	ISCED level 6: Bachelor’s or equivalent, vocational	ISCED level 5: Short-cycle tertiary education, vocational	ISCED level 4: Post-secondary non-tertiary, vocational	ISCED level 3: Upper secondary, vocational
Dementia screening programme/“case finding”: Carried out in primary care with the aim of detecting dementia at early stages. Different assessment tools might be used.	1					
	2	GP	RN			
	3					
	4	GP	RN dem			
	5	GP, MD psych,	RN, RN dem			
	6					
	7	GP, MD ger	RN, RN dem, RN distr, OT		LPN	
	8	GP, OT				
	9	GP				
Standard diagnostic procedure for dementia disease: National standard diagnostic procedure applied in accordance.	1	GP, MD ger, Psychologist	RN dem, RN distr, OT, SW			
	2	GP	RN distr			
	3	GP	RN, RN dem, RN comm			
	4					
	5	GP, MD psych,	RN dem, RN distr			
	6	GP	RN			
	7	GP	RN, RN distr, OT			
	8	GP				
	9	GP	OT			
Pharmacological treatment specific for dementia disease: Medication of cognitive symptoms, for instance Cholinesterase Inhibitors for improvement of cognition (memory, language). ATC code N06D [30].	1	GP, MD ger				
	2	GP	RN			
	3	GP, MD psych, MD neuro				
	4	GP	RN			
	5	GP, MD psych,				
	6	GP				
	7	GP				
	8	GP				
	9	GP				
Non-pharmacological treatment: Treatment – not pharmacological, for instance orientation to reality, reminiscence therapy, tactile massage, cognitive stimulation etc.	1				LPN, LPN dem	
	2		OT		LPN, LPN activity	
	3				LPN	SW-Ass
	4		RN, RN dem, SW, OT, PT		LPN	
	5		RN, RN dem, SW		LPN	
	6		RN, RN dem, RN distr, SW		LPN	
	7				LPN	
	8				LPN	
	9					
Pharmacological treatment specific for Behavioural Psychological Symptoms of Dementia (BPSD): Pharmacological treatment of non-cognitive symptoms and behaviours, psychotropic drugs for aggressive behaviour [31]. ATC code N05 [30].	1	GP, MD ger.				
	2		RN, RN-distr, RN-psych.			
	3	GP, MD psych.				
	4	GP, MD ger.	RN-dem, SW			
	5	GP, MD psych,				
	6	GP				
	7	GP				
	8	GP				
	9	GP				
Non-pharmacological treatment specific for BPSD: Treatment - not pharmacological, for instance environmental modification, massage, presence of pets, selected music, distraction etc. [23].	1		RN, RN-dem, dist, elderly		LPN, LPN activity.	
	2				LPN, LPN activity.	Ass-N
	3		OT		LPN, LPN activity.	Ass-N
	4	GP, MD ger.	RN-dem, SW, OT, FT			
	5		RN, RN-dem, RN-psych.			
	6		RN, RN-dem, −dist, SW, OT		LPN	
	7				LPN	
	8				LPN	
	9				LPN	Ass-N
Memory clinic: Outpatient clinic for examination and treatment of memory impairments, not only suspected dementia diseases.	1	MD ger, Psychologist	RN dem, OT, SW			
	2					
	3	MD psych,	RN, RN-psych			
	4					
	5	MD psych,	RN-psych			
	6	GP, MD ger, MD psych,	RN, RN-psych		LPN	
	7	MD psych, Psychologist	RN-psych, OT			
	8	GP				
	9	MD ger.				

*ISCED* International Standard Classification of Education [11]

For abbreviations of professional titles and qualifications, see Table 2

**Table 8:** Professional care providers level of education in the category *Outpatient care facilities*

		ISCED level 7: Master’s or equivalent, vocational	ISCED level 6: Bachelor’s or equivalent, vocational	ISCED level 5: Short-cycle tertiary education, vocational	ISCED level 4: Post-secondary non-tertiary, vocational	ISCED level 3: Upper secondary, vocational
Outpatient clinic specific for dementia diseases: Outpatient clinic in primary health care or in hospital, including care by physicians and nurses specialized in dementia.	1					
	2	GP	RN-distr			
	3	GP	RN-dem			
	4					
	5					
	6					
	7	GP	RN, RN-dem, RN-psych, OT			
	8		RN-distr,			
	9					
Specialized team in primary care A team specifically for people with dementia with different professionals; medical doctors, registered nurses, occupational therapists, physiotherapist and licensed practical nurses.	1	GP, Psychologist	RN-distr, OT			
	2	GP	RN-distr			
	3		RN, RN-dem			
	4					
	5					
	6	GP	RN		LPN	
	7					
	8	GP	RN, RN-dem, OT			
	9	GP	OT			

*ISCED* International Standard Classification of Education

For abbreviations of professional titles and qualifications, see Table 2

**Table 9:** Professional care providers level of education in the category *Institutional care*

		ISCED level 7: Master’s or equivalent, vocational	ISCED level 6: Bachelor’s or equivalent, vocational	ISCED level 5: Short-cycle tertiary education, vocational	ISCED level 4: Post-secondary non-tertiary, vocational	ISCED level 3: Upper secondary, vocational
Rehabilitation in institution: Training in residential home/nursing home/group dwelling by Occupational therapist, Physiotherapist and/or registered nurses and/or Licensed practical nurse/Auxiliary nurse to improve or maintain functional ability.	1		OT		LPN	Ass-N
	2		OT, FT		LPN, LPN-dem	
	3		OT, FT		LPN, LPN-dem	
	4		OT, FT		LPN	Ass-N
	5		OT, FT		LPN	
	6		OT, FT		LPN	
	7		OT, FT		LPN	Ass-N
	8		OT			
	9		OT		LPN	Ass-N
Safety accommodation: Accommodation for people 70+, not specifically for people with dementia. The accommodation is staffed specific hours every day to offer joint meals and activities.	1				LPN, LPN-dem	Ass-N
	2					
	3				CC	
	4				CC	
	5					
	6					
	7					
	8					
	9					
Residential home/Sheltered home/Assisted living. For older people not specific for those with dementia disease or mixed with other older people: Housing with care and service, for people who do not need daily attention from registered nurses or 24 h surveillance.	1		RN, RN-dem, SW,		LPN, LPN-dem	Ass-N
	2	GP	RN distr, RN psych, SW		LPN, LPN act, CC	Ass-N
	3		RN + dem, distr, elderly, SW		LPN, LPN-dem, CC	
	4		RN, OT, FT		LPN	
	5	GP	RN-dem, RN distr, FT			
	6	GP	RN, RN distr, SW		LPN	
	7		RN, RN-dem, SW		LPN	Ass-N
	8		SW			
	9		SW		LPN	Ass-N
Nursing home. Not specifically dementia disease or mixed with other older people: Nursing care 24/7, employed staff. May include short-term rehab, long-term care for people with chronic impairment or disabilities requiring daily attention of registered nurses to help with personal care or mobility.	1		RN, RN-dem, SW,		LPN, LPN-dem	
	2	GP	RN-distr, psych, elderly, SW, OT, FT		LPN, LPN-act, CC	Ass-N
	3		RN, RN-dem, distr, eld, SW		CC	
	4		RN, SW, OT, FT		LPN,	
	5					
	6					
	7		RN, RN-distr, SW		LPN	Ass-N
	8		SW			
	9				LPN	Ass-N
Nursing home with dementia care units: Nursing homes especially designed and adapted environment to meet the needs of people with dementia. Nursing care available 24/7 provided by employed staff. Units with staff specialized in dementia	1		RN-, RN-dem, elderly, SW		LPN, LPN-dem,	
	2					
	3					
	4					
	5					
	6					
	7		RN, RN-distr, SW		LPN	Ass-N
	8		SW			
	9					
Community Dwelling / Small housing for people with dementia: Housing especially designed and adapted environment to meet the needs of people with dementia. Staff specifically trained in dementia care. Only for people with dementia (≤10 people)	1		RN-, RN-dem, SW		LPN, LPN-dem	Ass-N
	2	GP	RN, RN--distr, OT, FT		LPN, LPN-act	Ass-N
	3					
	4					
	5	GP	RN-, RN-dem, SW		LPN	
	6					
	7					
	8					
	9					
Psychogeriatric unit/Geriatric psychiatry inpatient unit: Units where people with dementia are provided inpatient care, short-intermediate period of time (weeks to some months) with the aim of controlling acute BPSD. Staff specialized in dementia management	1					
	2					
	3					
	4					
	5	MD-psych				
	6					
	7					
	8	GP				
	9					

*ISCED* International Standard Classification of Education

For abbreviations of professional titles and qualifications, see Table 2

**Table 10:** Professional care providers level of education in the category *Palliative care*

		ISCED level 7: Master’s or equivalent, vocational	ISCED level 6: Bachelor’s or equivalent, vocational	ISCED level 5: Short-cycle tertiary education, vocational	ISCED level 4: Post-secondary non-tertiary, vocational	ISCED level 3: Upper secondary, vocational
Hospice/Palliative care at home: Hospice is a type of care and a philosophy of care, which focuses on the palliation of a terminally ill patient’s symptoms. The focus should be on physical, emotional, spiritual or social needs [32]. The care is provided at home.	1	GP, MD-ger	RN, RN-distr		LPN, LPN-dem	Ass-N
	2	GP	RN-distr, OT		LPN, OT-ass	Ass-N
	3		RN, RN-distr		LPN	Ass-N
	4		RN			
	5		RN-dem, RN-distr, SW		LPN	
	6	GP	RN, RN-distr, SW, OT, FT		LPN	
	7	GP	RN, RN-distr		LPN	
	8					
	9		RN			
Hospice/Institutional palliative care: Hospice is a type of care and a philosophy of care, which focuses on the palliation of a terminally ill patient’s symptoms. The focus should be on physical, emotional, spiritual or social needs [32]. The palliative care is provided in hospitals or nursing homes	1	GP, MD-ger	RN-, RN-dem		LPN, LPN-dem	Ass-N
	2	GP	RN, RN-distr, OT, FT		LPN	Ass-N
	3		RN, RN-distr,		LPN	Ass-N
	4		RN			
	5	GP	RN-dem, RN-distr, SW		LPN	
	6	GP	RN, RN-dem,/distr, SW, OT, FT		LPN	
	7	GP	RN, RN-distr		LPN	
	8					
	9		RN			
Advanced directive: Living will and power of attorney	1	GP, MD-psych	RN-, RN-elderly/distr			
	2		RN-, RN-distr			
	3					
	4					
	5					
	6					
	7					
	8					
	9					

*ISCED* International Standard Classification of Education [11]

For abbreviations of professional titles and qualifications, see Table 2

### Screening, the diagnostic procedures, and treatment

The care category *Screening, the diagnostic procedures, and treatment*, involved identification and treatment of dementia diseases and included the dementia screening programme, standard diagnostic procedure for dementia diseases and memory clinics as well as pharmacological and non-pharmacological treatment for dementia and assessment of behavioural psychological symptoms of dementia (BPSD) (Table 3). Standard diagnostic procedure and pharmacological treatment for specific dementia diseases was available for all and utilized by most people with a suspected dementia disease, predominantly in the early and intermediate stage. There was availability of pharmacological treatment also for BPSD but less utilization. Non-pharmacological treatments for specific dementia diseases such as orientation to reality, reminiscence therapy, tactile massage, and cognitive stimulation were available in almost all municipalities and most of these treatments were utilized in the intermediate stage. Non-pharmacological treatment for BPSD, such as environmental modification, massage, presence of pets, selected music, and distraction was available in more than half of the municipalities and was mostly utilized in the intermediate stage. Dementia screening in primary care and memory clinics were less available throughout all stages of the dementia disease. Most people utilized screening (where this was available) in primary care in municipalities; by contrast, memory clinics were utilized by few or no one.

### Outpatient care facilities

*Outpatient care* facilities included outpatient clinics specific for dementia and also specialized teams in primary care. Specialized teams in primary care may be necessary depending on the needs of the person with dementia and were more common than outpatient clinics for people with dementia. Where available, they were utilized by most in the early to intermediate stage (Table 4).

### Institutional care

The care category *Institutional care* encompassed institutional rehabilitation, institutions or residential homes, and temporary or planned as well as specialized or not specialized care. Institutional rehabilitation and residential homes were available for all, but municipalities differed in utilization. Nursing homes for older people in general were available in more than half of the municipalities with variation regarding utilization. All the care activities under this category were utilized by most, predominantly in the late and end of life stage (Table 5). Availability of nursing homes and community dwellings with dementia care units as well as psychogeriatric units was rare in the municipalities and where they existed, they were utilized by most or all.

### Palliative care

The care category *Palliative care* encompassed care at home, institutional care and advanced directive services. Home and institutional care was available for all in most of the municipalities through all stages of the dementia disease, but was utilized by few (Table 6). In one municipality, palliative care was available for no one. Advanced directive services were available for all in two municipalities through all stages of the dementia disease and for few or no one in the remaining seven municipalities. Where available, they were utilized by few except in the end of life stage.

### Professionals providing care and their educational level

Professionals providing the care in the care category *Screening, the diagnostic procedures, and treatment* most often held a Bachelor’s degree to a Master’s degree in Science (Table 7). Professionals holding a post-secondary qualification or a Bachelor’s degree provided non-pharmacological treatment. Professionals in some municipalities were specialized in dementia care, most often in the standard diagnostic procedure for dementia diseases or in memory clinics. *Outpatient care facilities* in several municipalities engaged professionals holding a Bachelor’s or a Master’s degree (Table 8). In one municipality, professionals held a post-secondary education; professionals specialized in dementia care were rare. Professionals involved in *Institutional care* in residential homes, nursing homes and community dwellings held a post-secondary educational level, a Bachelor’s, or Master’s degree (Table 9). Professionals specialized in dementia care most often worked in residential homes. *Palliative care* was provided by professionals educated to a post-secondary educational level or holding a Bachelor’s or Master’s degree, some of whom were specialized in dementia care (Table 10).

## Discussion

The previously developed mapping system for dementia care was found to be reliable after making minor adaptation to the context at the local level, i.e. the nine municipalities studied. The categories developed were found to be robust and no changes were needed; however slight adaptations to definitions were needed. Adaptation of activities in the different municipalities was also needed. The differences between estimations of availability and utilization in the diagnosis stage and early stage were insignificant the two stages were collapsed into one stage, the “early stage”. Care activities were added and removed to fit the local context although not many alterations were made. Furthermore, minor adjustments were made to the definitions of care activities through the course of the dementia disease.

The results revealed a range of different care activities, more than contained in the original mapping system. These results suggest that the mapping system is a valid tool for exploring and comparing care and service systems for persons with dementia and their caregivers at a local level. However adaptations may be needed in terms of care activities offered and their definition. Further, the results revealed differences between the nine municipalities and allowed comparison between municipalities that are responsible for the care of people with dementia, also making the options for care and support visible. It is well known that people with dementia and their caregivers have individual needs throughout the stages of dementia. Some have reported perceiving health care professionals as reactive rather than proactively informing the patient and their caregiver(s) about the disease progression and the support available to meet their needs [5]. By systematically exploring the available care activities, professionals and policy makers could be proactive in their communication with care recipients and fill gaps where needed. In order to further bring light on what is needed throughout the course of dementia it would be worthwhile to interview the informal caregivers and the person with dementia to find out what they know about options throughout the disease progression.

Persons with dementia in the nine municipalities do not all seem to have equal access to care and services despite the existing national or local guidelines for dementia care. For instance, in this study, standard diagnostic procedure for dementia diseases was available in all municipalities except one. In addition, there were differences between municipalities regarding dementia screening carried out in primary care and memory clinics. This might relate to availability of competence in the primary health care centres but also to adherence to guidelines which shows a diversity between the physicians’ length of clinical experience and among patients [21]. Pharmacological treatment for dementia diseases was available for all, which is consistent with the results from Swedish national data [1, 2] and utilized by most. Pharmacological treatment for BPSD was available for all, but on the whole it was utilized by few which is in accordance with national directives [22]. Non-pharmacological treatment of BPSD, such as environmental modification, distraction, massage, presence of pets and listening to selected music [23], which should be easy to apply for professionals and which has few or no side effects, was available in six municipalities, where it was utilized by most in the intermediate to late stage. This was inconsistent with the RTPC study [1] where non-pharmacological treatment was available through all stages of the dementia disease. Outpatient care facilities in the present study were few and not often used, regardless of rural or urban location. Differences were found also between municipalities regarding institutional care specifically for people with dementia. This was available in fewer than half of the municipalities, regardless of rural or urban location. By contrast, in the end stage of life, palliative care was available in close to all municipalities with mixed utilization and with no differences between municipalities. The quality of these care and treatment activities cannot be revealed by this study; however, by making the differences visible the study may initiate a process of discussing and developing a system based on the needs of the patients and their informal caregivers.

In accordance with the RTPC study [1] the present study shows differences between availability and utilization of care activities. Several care activities were available for all or most people with dementia, but utilized by few or no one, with some variation between municipalities and care categories. For instance, in the early to intermediate disease stage when diagnosis and treatment is needed, utilization was more frequent in the category *Screening, the diagnostic procedures, and treatment*. The modest availability of *Outpatient care facilities*, on the other hand may be due to smaller municipalities using the outpatient care facilities in larger municipalities. When the dementia disease progresses towards late and end of life stage, and the person with dementia has extensive care needs, there is an increase in utilization of institutional care and institutional palliative care.

There may be several explanations for non-utilization of available care activities in this study. One likely explanation is that the informal caregiver(s) and the care recipient may not be aware of the care and service activities that are available. It may also be that the care activities are not convenient or judged too expensive and/or too far away [10]. The visibility of the care is probably the most important explanation and this may coincide with a gate-keeper attitude. It has been shown that the system has been experienced as fragmented, with different professional providers and organizations responsible for different care and service activities, resulting in uncertainties regarding what to ask for and whom to contact within the care system [6, 7]. At present, a coherent chain of care exists for cancer care in Sweden, but not yet for dementia care [24]. By systematically assessing availability of services and utilization, we can learn valuable knowledge about the local health care and social service system, which can form the basis for developing needed care activities, eliminating any that are not needed and communicating with those in need of support. It will make the chain of care visible and enable development of the existing health care and social service system at a local level for policy makers and professionals. This is essential to avoid a gate-keeper situation and instead ensure transparency, accessibility, and equal and individualized health care and social services for dementia care. Timely diagnosis and timely access to care can help people with dementia and their caregivers in their care planning and enable them to take control over their situation [2].

The health professionals’ educational level varied between the different care activities and municipalities, which may have affected the ability to provide high-quality care. Professionals with the lowest level of education were those involved in the everyday care of the persons with dementia and their caregivers in home and institutional palliative care, which is in line with findings from the European study [14]. For instance, those providing non-pharmacological treatment, held an upper-secondary ISCED qualification [11] while professionals working in institutional and palliative care were educated to upper-secondary ISCED level or above. The consequences of low professional education in the intimate care activities can be debated; this could for instance be detrimental for the quality of care but also for the staff’s work environment. Professionals involved in screening, diagnosis and outpatient care held a Master’s or in some cases, a Bachelor’s degree. This is similar to the results reported from the RTPC study [14]. With an ageing population, the demands on long-term services will increase. To meet these demands, highly skilled staff are needed. It has been suggested that, to increase the quality of care and residents’ quality of life, the competences of baccalaureate-educated registered nurses are needed [25].

Specialized training in dementia existed in the care categories analysed in this study, predominantly involving professionals with a Bachelor’s degree but also those with an upper-secondary ISCED level education, which is inconsistent with the RTPC data [14]. This may be due to the national guidelines for dementia care. Also there is awareness among the public that professionals specialized in dementia care are able to provide individualized care throughout the course of the dementia disease [15]. Dementia specific education existed at different levels, from upper-secondary ISCED to Master’s degree level. Specialized training in dementia was rare in this study, and existed in fewer than half of the municipalities. Notable was that professionals specialized in dementia care were involved in the care of elderly patients in general, indicating that, in these municipalities, it was considered important to be able to care for people with dementia at general facilities. It is essential that professionals providing health care and social services to persons with dementia and their informal caregivers have relevant education and knowledge so that they can provide dementia-specific, individualized care and services [26, 27]. Research is sparse and further research is needed regarding whether specialized training in dementia has an impact on the quality of care for people with dementia living at home and in nursing homes [25, 28].

This study has limitations. We are only starting to investigate the system of care and services for a severe disease like dementia. It may be that the response alternatives for availability and utilization were too vague. Also the data were collected during April and May 2015 and it is possible that data would be different if collected today. Attempts were made to define each care activity; however, the descriptions are still open to interpretation, e.g. by the professionals. To address this problem, those performing the data collection were given specific training and instructions. The quality of the care activities cannot be stated in this study although some indications depending on the educational level of those involved can be seen. Also, the reasons for differences between availability and utilization needs to be further explored and so also does how the options in the system are communicated to those who might need it. Furthermore, no data were collected on how responsibility for care and services was distributed in real life. For people with dementia and their caregivers, it is of utmost importance to know who is responsible for which care activities and who to turn to. The mapping system can, and needs to, be adapted to each heath care and social service system and to national guidelines where available. To strengthen the mapping system for interconnection, descriptions of care activities should be more specific to avoid interpretation by professionals. Non-uptake of services and additional needs and the link between these may have to be studied further by additional interviews or surveys to get an idea of the complexities of the chain of care.

The strength of this study was that each municipality was given the same instructions for data collection, ensuring reliability. A pilot test of the developed mapping system was performed after re-fining descriptions of care activities and providers. However, each contact person reported availability and utilization of municipality care and services, as well as providers’ educational level, from a local perspective, which may have affected the validity of the results. Testing the mapping system at a local level, regarding availability and utilization of care activities and professionals educational level, and further, comparing local data between municipalities worked well, thus revealing differences that suggest that the mapping system is an instrument able to assess and intercept variation.

## Conclusions

A previously developed mapping system for investigating resources in health care and social services for people with dementia and their informal caregivers was adapted and tested in nine municipalities in Sweden to enable its use at a local level. Minor context-related changes were made regarding care activities and professionals’ educational level. The categories capturing the type of care activities available was found to be reliable. The system enables professionals and policy makers to reveal strengths and weaknesses in the health care and social services system when providing care and services on equal terms for people with dementia and their informal caregivers. Mapping the educational level of professionals providing the care and services may reveal, where in the chain of care, dementia-specific education for professionals needs to be developed. Further, the mapping system may enable professionals to be proactive and communicate available care and services to people with dementia and their caregivers throughout the course of the dementia disease. This is also invaluable in policymaking.
